# Supplementary material for: An all-in-one pipeline for the in vitro discovery and in vivo testing of Plasmodium falciparum malaria transmission blocking drugs
Source: Nat Commun. 2025 Jul 25;16:6884. doi: 10.1038/s41467-025-62014-3 (PMC12297408; doi:10.1038/s41467-025-62014-3)
Supplement: Supplementary file 1 — Supplementary Information [file 41467_2025_62014_MOESM1_ESM.pdf]

Supplementary Information

An all-in-one pipeline for the *in vitro* discovery and *in vivo* testing of *Plasmodium falciparum* malaria transmission blocking drugs

Nicolas M. B. Brancucci*, Christin Gumpp, Geert-Jan van Gemert, Xiao Yu, Armin Passecker, Flore Nardella, Basil T. Thommen, Marc Chambon, Gerardo Turcatti, Ludovic Halby, Benjamin Blasco, Maëlle Duffey, Paola B. Arimondo, Teun Bousema, Artur Scherf, Didier Leroy, Taco W. A. Kooij, Matthias Rottmann*, Till S. Voss*

*Corresponding authors. Email: till.voss@swisstph.ch (TSV), matthias.rottmann@swisstph.ch (MR), nicolas.brancucci@swisstph.ch (NMBB)

THIS SUPPLEMENTARY INFORMATION FILE INCLUDES

Supplementary Materials and Methods

Figs. S1 to S8

Table S1 to S2

Data files S1 to S5

Supplementary Materials and Methods

Workflow for automated image analysis of Hoechst-/MitoTracker-stained stage V gametocytes.

Step 1	Setup		
		Image Names:	Channels:
		Hoechst	HOECHST
		MitoTracker	TRITC

This step defines the channels used for analysis.

Step 2	Find Blobs		
		Source	MitoTracker
		Approximate Minimum With (µm)	2
		Approximate Maximum With (µm)	20
		Intensity Above Local Background	300
		Result	MitoTracker signal

This step identifies signals above local background fluorescence in the TRITC channel. Note that the cytoplasm of gametocytes, rather than the mitochondrion only, is included in the "MitoTracker signal" mask (result) using this relatively low intensity threshold. Compare comments in steps 4 and 5.

Step 3	Remove Boarder Objects		
		Source	MitoTracker signal
		Result	Mitochondria w/o border objects

This step removes objects situated at the image border of the "MitoTracker signal" mask.

Step 4	Filter Mask		
		Objects Source	Mitochondria w/o border objects
		Marker Source	Mitochondria w/o border objects
		Measurement	Total Area
		Filter Type	Min Filter
		Maximum Value	15
		Include Min/Max Values	No
		Measurement	Ell. Form Factor
		Filter Type	Min Filter
		Maximum Value	1.8
		Include Min/Max Values	No
		Result	Mitochondria ell. form factor

This step removes MitoTracker signal falling below a minimum area and crescent shape (compare comments in step 2) from living cells.

Step 5	Find Blobs		
		Source	MitoTracker
		Approximate Minimum With (µm)	1
		Approximate Maximum With (µm)	10

		Intensity Above Local Background	2500
		Result	Mitochondria intensity threshold

Using a high intensity threshold for MitoTracker signals, this step identifies mitochondria of living gametocytes.

Step 6	Find Round Objects		
		Source	Hoechst
		Approximate Minimum With (μm)	0.8
		Approximate Maximum With (μm)	3
		Intensity Above Local Background	250
		Result	Round Hoechst objects

This step identifies parasite-infected red blood cells based on the DNA dye Hoechst.

Step 7	Grow Objects		
		Objects Source	Round Hoechst objects
		Grow by	8
		Result	Nuclei grown

This step grows the "Round Hoechst objects" mask by 8 pixels in both dimensions.

Step 8	Remove Border Objects		
		Objects Source	Nuclei grown
		Result	Nuclei w/o border objects

This step removes objects situated at the image boarder of the "Nuclei grown" mask.

Step 9	Find Blobs		
		Source	Hoechst
		Approximate Minimum With (μm)	100
		Approximate Maximum With (μm)	300
		Intensity Above Local Background	89
		Result	Blobs

This step identifies background signal (large contaminants) in images acquired in the HOECHST channel.

Step 10	Grow Objects			
		Objects Source		Blobs
		Grow by		200
		Result		Blobs grown

This step grows the "Blobs" mask by 200 pixels in both dimensions.

Step 11	Remove Marked Objects		
		Objects Source	Nuclei w/o border objects
		Marker Source	Blobs grown
		Result	nuclei

This step removes areas with significant background from the "Nuclei w/o border objects" mask.

Step 12	Keep Marked Objects		
		Objects Source	Mitochondria ell. form factor
		Marker Source	nuclei
		Result	Gametocytes

This step identifies gametocytes within infected red blood cells.

Step 13	Keep Marked Objects		
		Objects Source	Gametocytes
		Marker Source	Mitochondria intensity threshold
		Result	Live gametocytes

This step identifies living gametocytes within the "Gametocytes" mask.

Step 14	Measure Mask		
	Objects to Measure	Mask of Objects	nuclei
		Image to Measure	nuclei
	Features within Each Object	Mask of Features	Mitochondria Ell. form factor
		Image to Measure	nuclei
	Features within Each Object	Source 1	Live gametocytes
		Source 2	Gametocytes

This step quantifies the number of viable gametocytes among infected red blood cells.

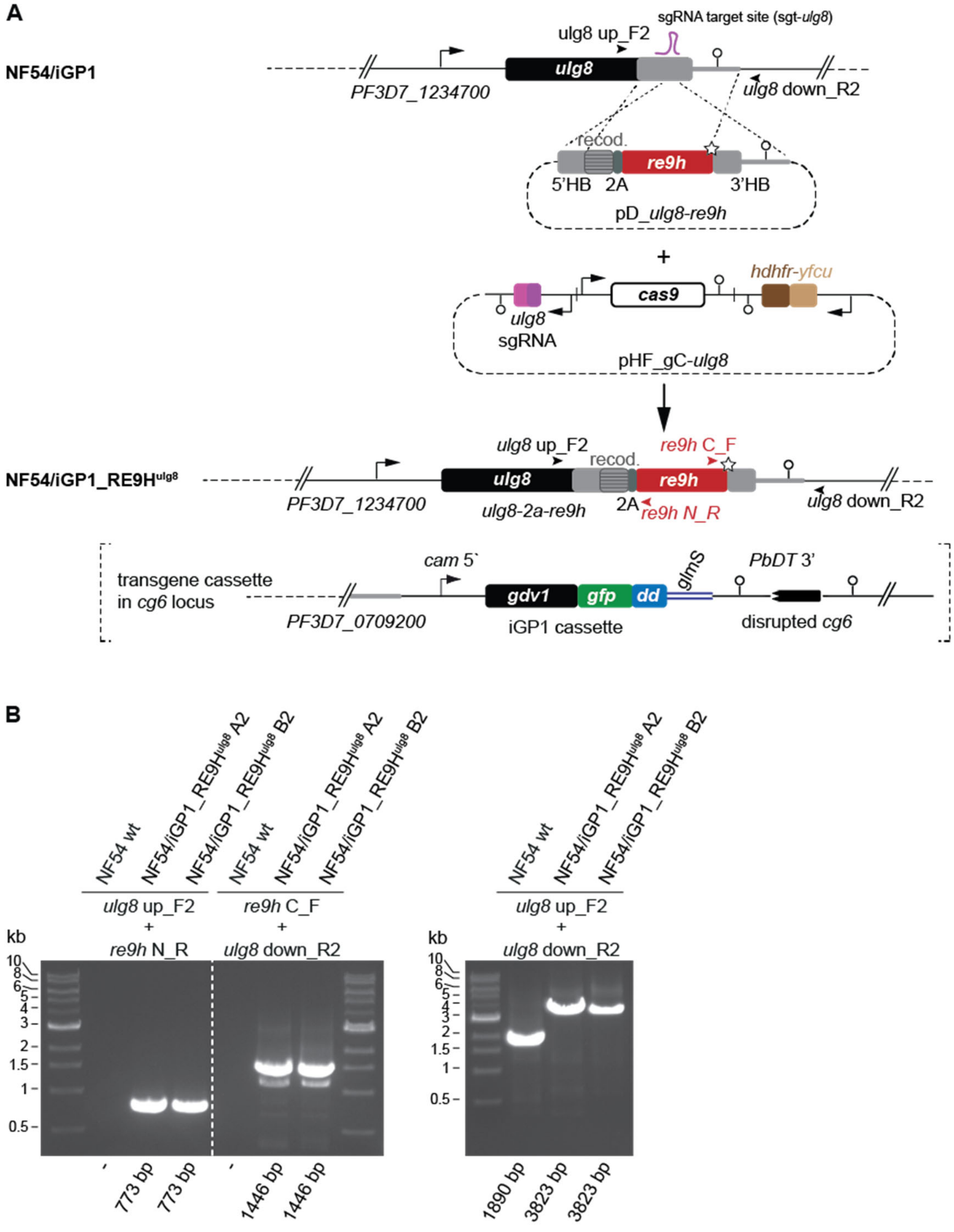

Fig. S1. Generation of the NF54/iGP1_RE9H^{ulg8} gametocyte reporter line. (A) Schematic map of the endogenous *ulg8* locus (PF3D7_1234700) (top), the pD_*ulg8_re9h* donor and pHF_gC-*ulg8* plasmids (center), and the gene-edited *ulg8* locus expressing a ULG8-2A-RE9H fusion (bottom) in NF54/iGP1_RE9H^{ulg8} parasites. The relative position of the sgt_*ulg8* sgRNA target sequence is shown in purple. The pD_*ulg8-re9h* donor plasmid contains the 5' and 3' homology boxes (HB) for homology-directed repair (light grey), flanking the re-codonized 3' end of the *ulg8* gene (shaded light grey) followed by sequences encoding the 2A split peptide (dark grey) and the RE9H luciferase (red). The STOP codon is indicated by a white star. The pHF_gC-*ulg8* plasmid contains a SpCas9 expression

cassette (white), the sgRNA expression cassette (pink-purple) and the *hdhfr-fcu* positive-negative drug selection marker cassette (brown-grey). Oligonucleotide binding sites used to confirm successful tagging of the *ulg8* gene by PCR on gDNA are indicated by arrowheads. The schematic in brackets at the bottom shows the inducible *gdv1-gfp-dd-glmS* over-expression cassette previously inserted into the non-essential *cg6* locus (PF3D7_0709200) in NF54/iGP1 parasites[71]. *cam* 5', *P. falciparum* calmodulin promoter; PbDT 3', *P. berghei dhfr-ts* terminator. glmS, glmS riboswitch element. (**B)** Results of diagnostic PCRs performed on gDNA from NF54 wild-type parasites (control) and two NF54/iGP1_RE9H^{ulg8} clones (A2 and B2). Primer combinations detecting the 5' and 3' recombination events are shown in the left panel. Primer combinations detecting the presence and absence of the *ulg8* wild-type locus in NF54 wild-type and NF54/iGP1_RE9H^{ulg8} parasites, respectively, and the presence of the *ulg8-2a-re9h* fusion gene specifically in NF54/iGP1_RE9H^{ulg8} parasites are shown in the right panel. NF54/iGP1_RE9H^{ulg8} clone B2 has been selected for further experiments.

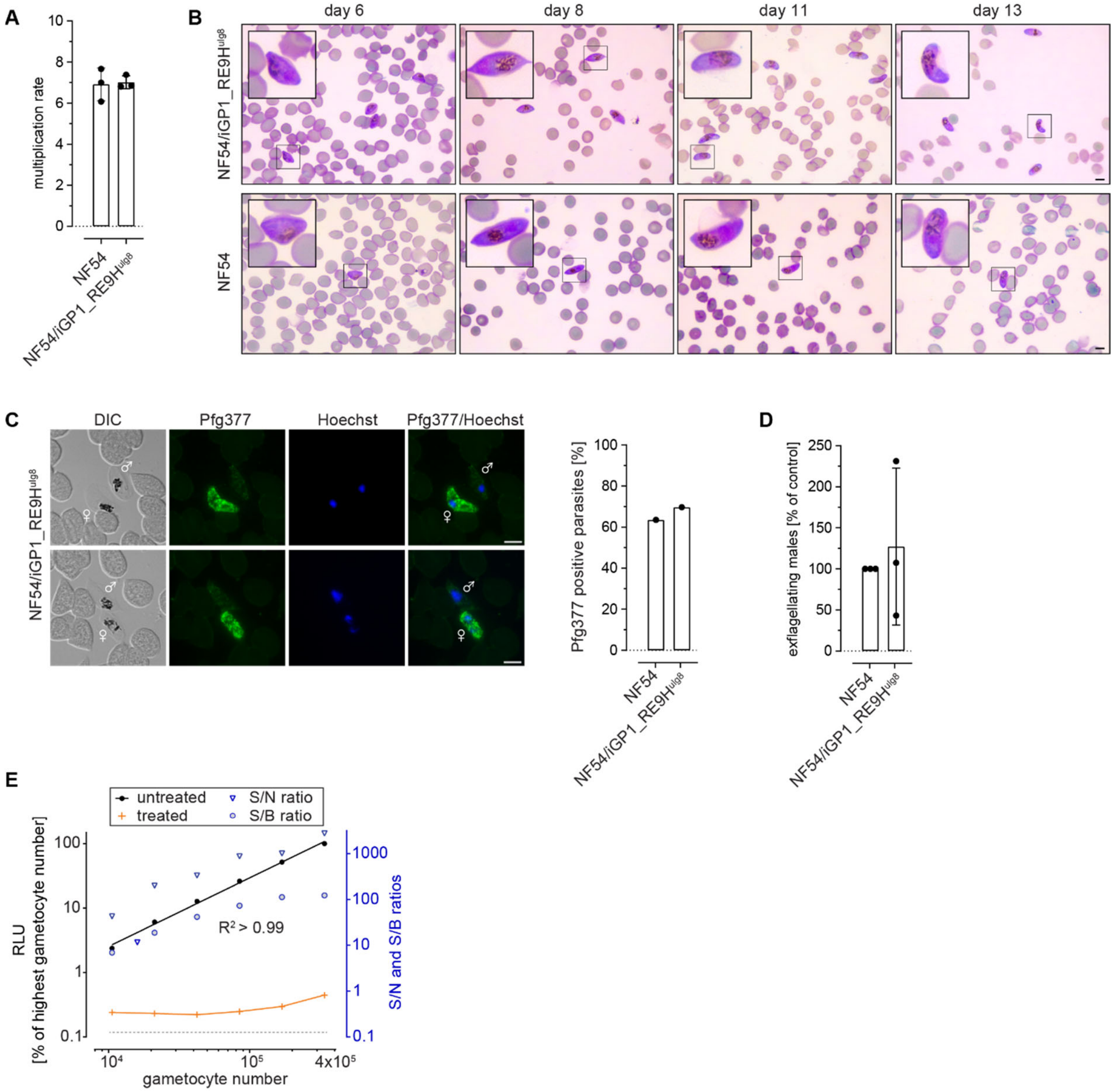

Fig. S2. Characterisation and validation of NF54/iGP1_RE9H^{ulg8} parasites as a suitable gametocyte reporter line. (A) Parasite multiplication rates of NF54/iGP1_RE9H^{ulg8} are comparable to those of the NF54 wild type control (mean ± s.d.) (n=3 biological replicates; individual values are represented as black dots). **(B)** NF54/iGP1_RE9H^{ulg8} gametocytes show the expected stage-specific morphology throughout development. Representative images of n=3 biological replicates are shown. Days of gametocyte development (days post invasion of sexually committed merozoites) are indicated. **(C)** NF54/iGP1_RE9H^{ulg8} and NF54 wild type gametocyte populations display similar female-biased sex ratios. Pfg377 expression in female stage V gametocytes was assessed by IFA. Representative images are shown (n=1 experiment). DNA was stained with Hoechst. Female (Pfg377-positive) and male cells (Pfg377-negative) are highlighted by symbols. Scale bar = 5 µm. The proportions of gametocytes positive for the female marker Pfg377 among all gametocytes are shown on the right (at least 200 gametocytes were counted per sample). **(D)** Exflagellation rates of NF54/iGP1_RE9H^{ulg8} stage

V gametocytes (day 13) are comparable to those obtained for the NF54 wild type control (mean ± s.d.) (n=3 biological replicates; individual values are represented as black dots). **(E)** RE9H-catalyzed bioluminescence signals obtained from a dilution series of NF54/iGP1_RE9H^{ulg8} stage V gametocytes (day 12; 3% hematocrit) either left untreated (black dots) or treated with 50 µM MB (orange crosses) for 72 hours. Values on the left y-axis represent RLUs normalized to the mean signal emitted from the wells containing the highest gametocyte number (mean of four technical replicates, n=1 experiment). The linear regression line (black) and the coefficient of determination (R^2) for untreated gametocytes are indicated. Values on the right y-axis represent the S/N ratios (blue triangles) and S/B ratios (blue circles) calculated based on the RLUs obtained from MB-treated and untreated gametocytes. The dashed line reflects the normalized RLUs measured from control wells containing uninfected RBCs.

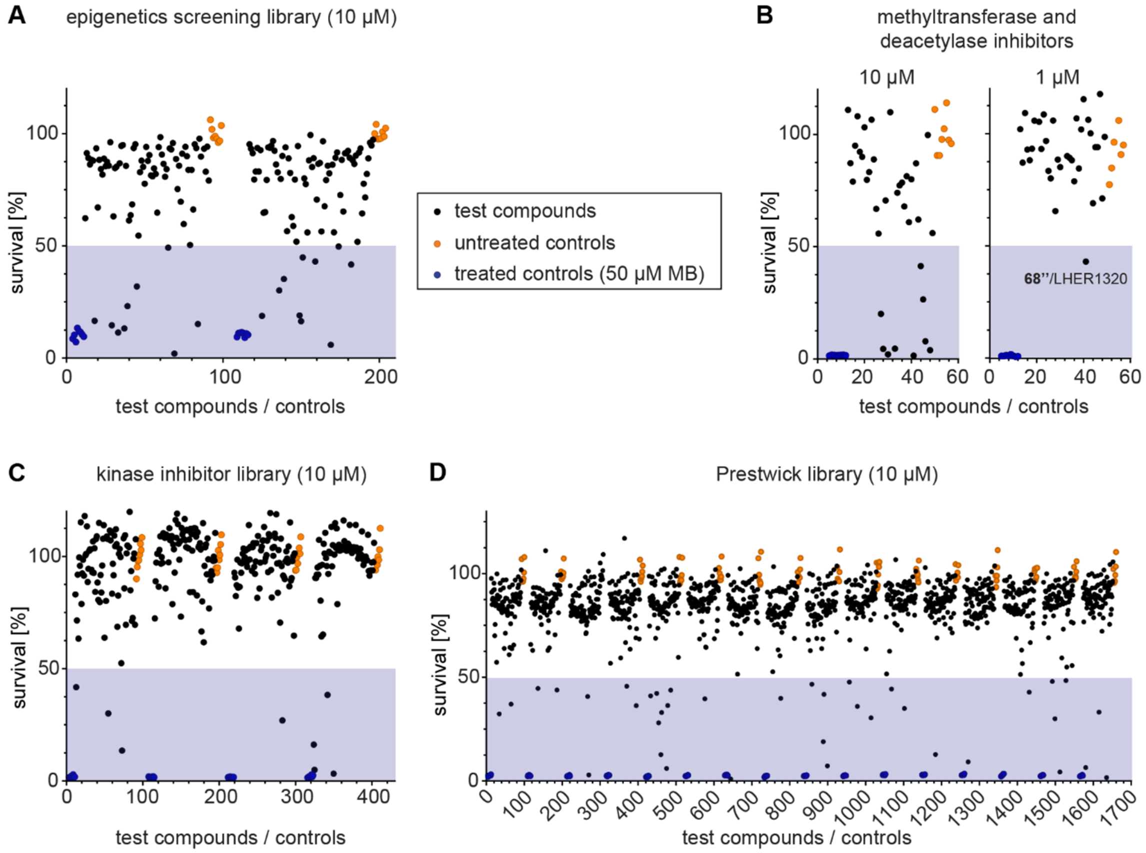

Fig. S3. Screening of chemical libraries against NF54/iGP1_RE9H^{ulg8} stage V gametocytes. Results from the primary screening of small- to medium-sized compound libraries. **(A-D)** Effect of compounds of the Epigenetics Screening Library (Cayman Chemical) (10 µM concentration) (A), inhibitors of human DNMTs, HKMTs and histone deacetylases (10 µM and 1 µM concentration) (compound **68''**/LHER1320 with >50% inhibitory activity 1 µM is highlighted), human kinase inhibitors (SelleckChem, Enzo Life Sciences) (10 µM concentration) (C) or compounds of the Prestwick Chemical Library (10 µM concentration) (D) on NF54/iGP1_RE9H^{ulg8} stage V (day 12) gametocyte viability. Each assay plate included eight treated (50 µM MB; blue dots) and untreated (0.1% DMSO; orange dots) samples each as positive and negative controls, respectively. Values on the y-axis represent RLUs normalized to the mean signal emitted from the untreated controls, obtained from n=1 experiment for each library. Compounds with >50% inhibitory activity are highlighted (blue shaded areas).

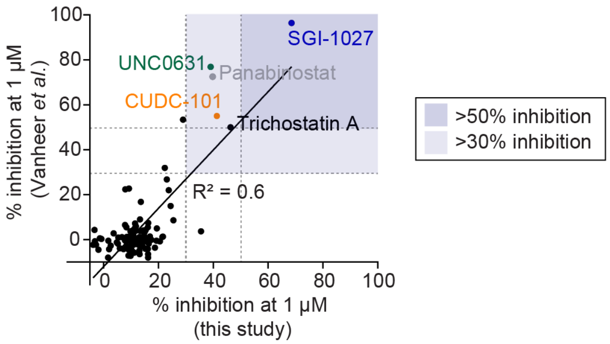

Fig. S4. Comparison of the gametocytocidal activities of compounds of the Epigenetics Screening Library (Cayman Chemical) screened here and by Vanheer et al. *(67)*. Scatter plot correlating the inhibitory effects on gametocyte viability of compounds targeting epigenetic enzymes screened at 1 µM concentration against late stage gametocytes (day 10; stage IV/V) in the Vanheer study[67] and against mature stage V gametocytes (day 12) in this study. Compounds showing >30% and >50% inhibition are highlighted.

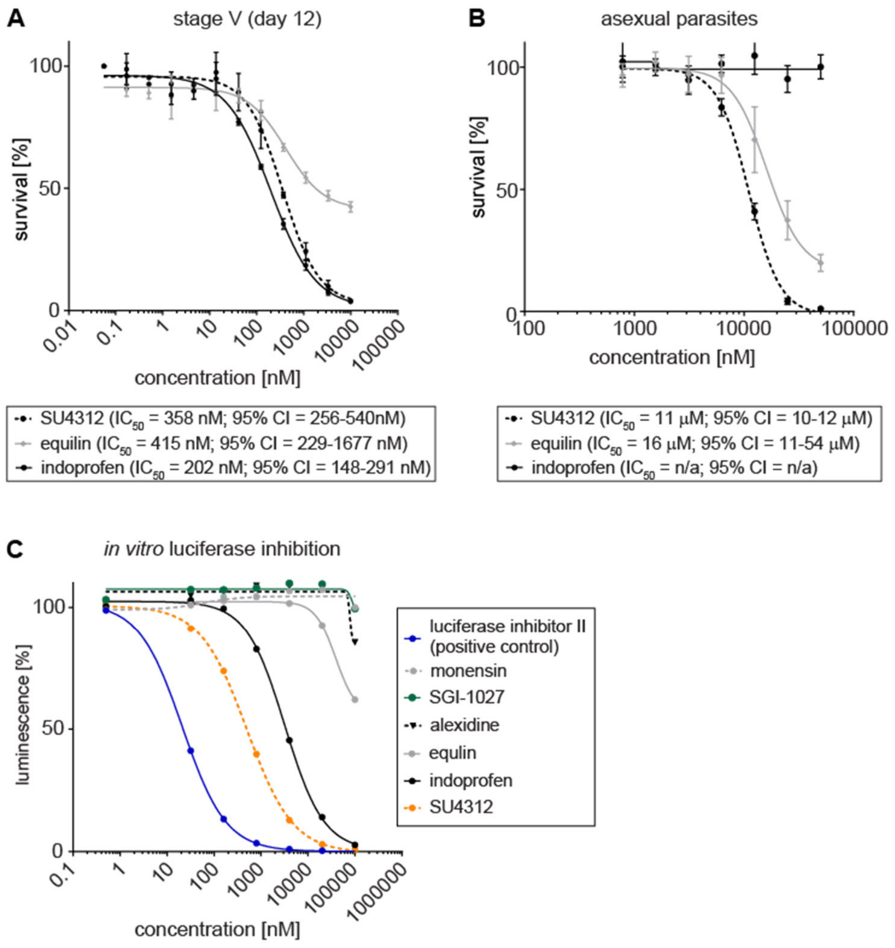

Fig. S5. Effect of hit compounds against NF54/iGP1_RE9H^{ulg8} stage V gametocytes, asexual parasites and *in vitro* luciferase activity. (A) Dose-response curves of SU4312, equilin and indoprofen tested against NF54/iGP1_RE9H^{ulg8} stage V gametocytes (day 12). Values on the y-axis represent RLUs normalized to the mean signal emitted from cells exposed to the lowest drug concentration, obtained from n=3 biological replicates (mean ± s.e.m.). **(B)** Dose-response curves of SU4312, equilin and indoprofen tested against NF54 wild-type asexual blood stage parasite multiplication. Values on the y-axis represent [^{3}H]-hypoxanthine incorporation normalized to the mean signal emitted from eight untreated control samples per plate, obtained from n=3 biological replicates (mean ± s.e.m.). IC_{50} values and 95% confidence intervals (CI) are shown below the graphs. **(C)** Dose-response curves of all six hit compounds tested for inhibition of RE9H luciferase activity *in vitro*. Values on the y-axis represent RLUs normalized to the mean signal obtained from four untreated control samples (mean of two technical replicates, n=1 experiment). Luciferase inhibitor II was used as a positive control.

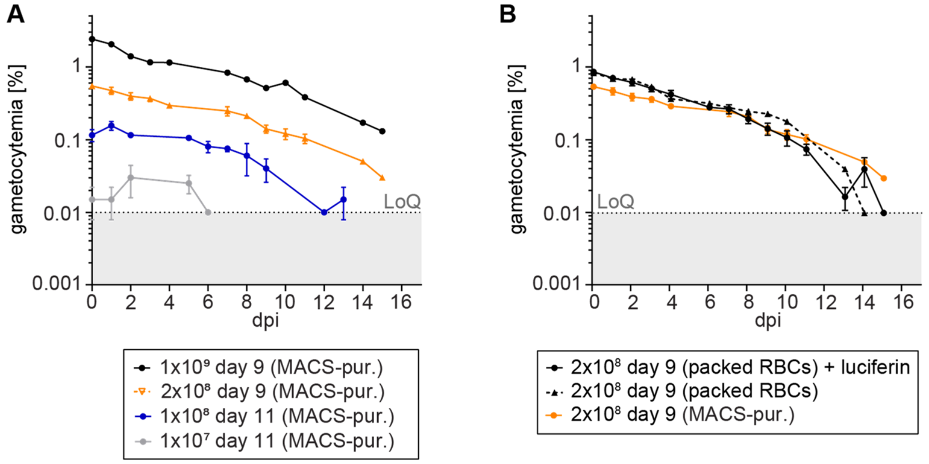

Fig. S6. Optimisation of the protocol used to infect hRBC-engrafted NODscidIL2Rγ^{null} mice with NF54/iGP1_RE9H^{ulg8} stage V gametocytes. Course of peripheral gametocytemia in NF54/iGP1_RE9H^{ulg8}-infected mice. **(A)** Mice were infected with 1x10^{7} (n=2 mice), 1x10^{8} (n=2 mice), 2x10^{8} (n=3 mice) or 1x10^{9} (n=1 mouse) MACS-purified NF54/iGP1_RE9H^{ulg8} gametocytes (day 9 or 11). **(B)** n=8 mice were infected with packed RBCs containing 2x10^{8} NF54/iGP1_RE9H^{ulg8} day 9 gametocytes obtained directly from *in vitro* cultures without prior purification, seven of which received daily injections of 150 mg/kg D-luciferin. Data from mice infected with 2x10^{8} MACS-purified NF54/iGP1_RE9H^{ulg8} day 9 gametocytes are identical to those shown in panel A. Values on the y-axis represent peripheral gametocytemia (mean ± s.d.) determined by microscopic inspection of Giemsa-stained thin blood smears prepared from tail blood 30 min after infection and once daily thereafter for 15 days. dpi, days post infection; LoQ, limit of quantification (area below the LoQ is shaded gray).

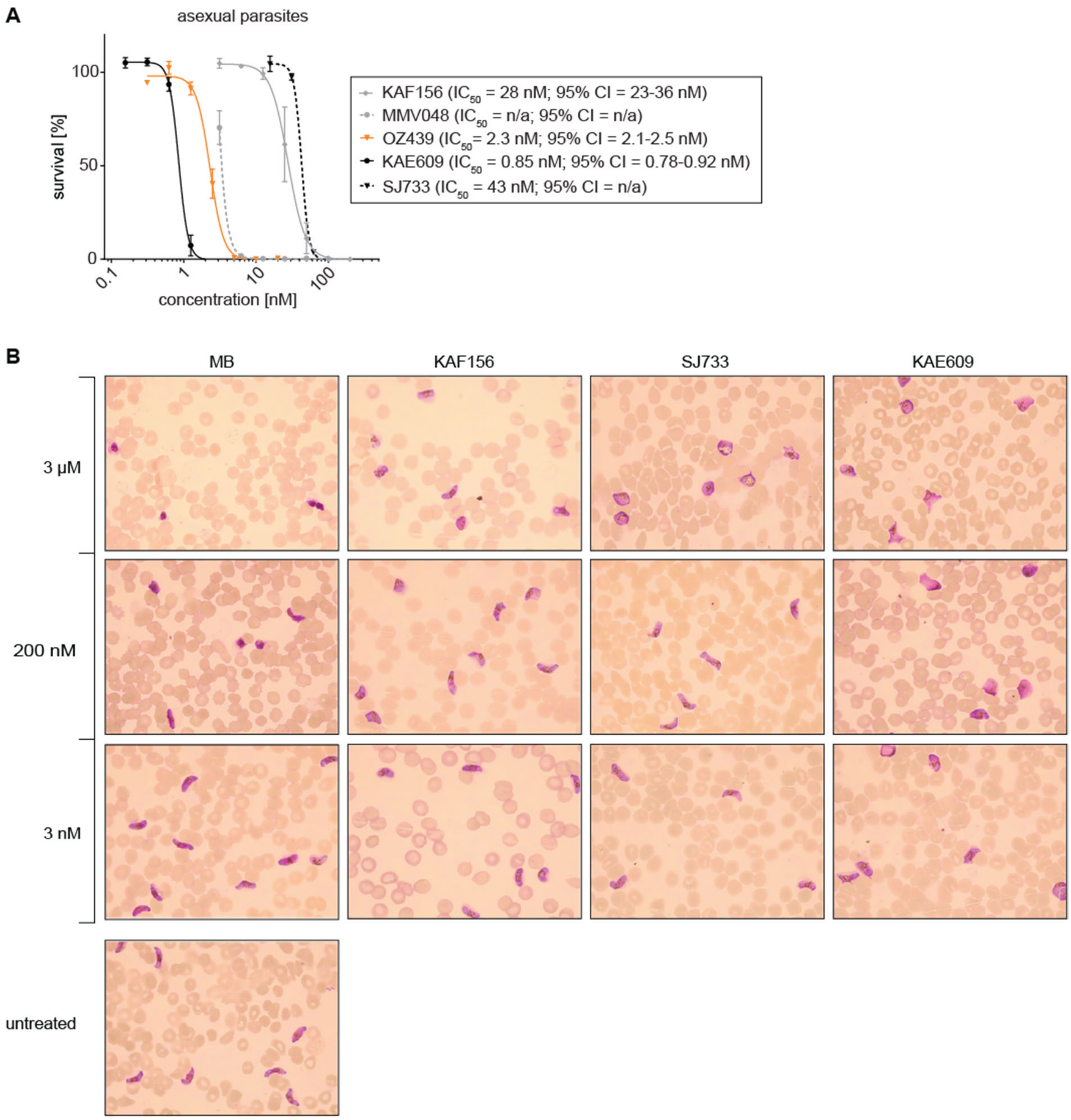

Fig. S7. Effect of clinical drug candidates on asexual parasite proliferation and stage V gametocyte morphology. (A) Dose-response curves of clinical drug candidates tested against NF54 wild-type asexual blood stage parasite multiplication. Values on the y-axis represent [^{3}H]-hypoxanthine incorporation normalized to the mean signal emitted from eight untreated control samples per plate, obtained from n=3 biological replicates (mean ± s.e.m.). IC_{50} values and 95% confidence intervals (CI) are shown on the right. Note that an IC_{50} value for MMV390048 could not be determined due to lack of data points in the sublethal concentration range. **(B)** Representative images taken from Hemacolor-stained thin blood smears prepared from NF54/iGP1_RE9H^{ulg8} stage V gametocytes (day 12) treated for 72 hours *in vitro* with different concentrations of MB, KAF156/ganaplacide, SJ733 and KAE609/cipargamin. Untreated gametocytes (day 15) are shown as control.

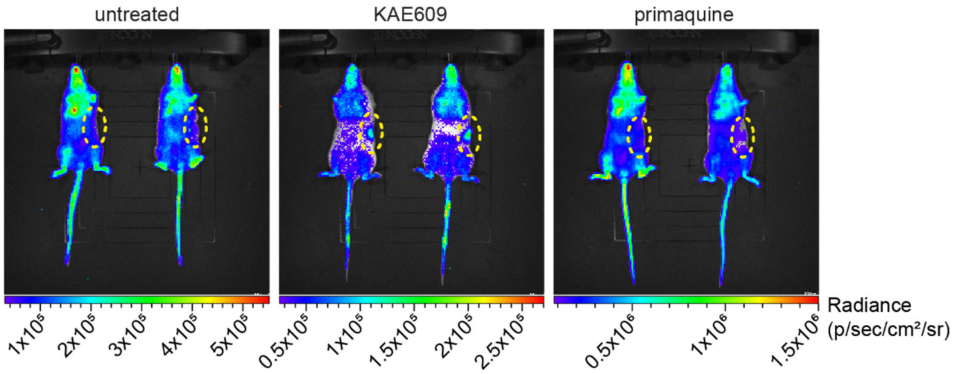

Fig. S8. Evidence for splenic retention of NF54/iGP1_RE9H^{ulg8} stage V gametocytes in KAE609/cipargamin-treated NSG-PfGAM mice. Representative ventral images of mice infected with 2x10^{8} NF54/iGP1_RE9H^{ulg8} stage V gametocytes (day 11) and treated one day after infection with 1x40 mg/kg KAE609/cipargamin or 1x50 mg/kg PQ (positive control) or left untreated. Images were recorded after i.v. injection of 150 mg/kg D-luciferin and anesthesia 24 hours post treatment. Pseudocolour heat-maps indicate RE9H-catalysed bioluminescence intensity from low (blue) to high (red). Automated scaling was applied for individual groups to enable comparison of gametocyte densities between heterogeneous groups (e.g. untreated mice with higher gametocyte load compared to low gametocyte load in treated groups). The dashed ovals demarcate the location of the spleen, indicating splenic retention of gametocytes upon treatment with KAE609/cipargamin (n=1 experiment).

Table S1. Stage-specific gametocytocidal activity of reference antimalarial drugs and experimental compounds.

	IC_{50} [nM (95% CI)] (this study)			IC_{50} (nM ± s.d.)[6]			
**Compound**	**III (day 5)**	**IV (day 8)**	**V (day 12)**	**II (day 4)**	**III (day 6)**	**IV (day 8)**	**V (day 12)**
Chloroquine	77 (n/a)	n/a	41,000 (n/a)	98 ± 5	>6,250	>6,250	>6,250
Artemisinin	42 (34-51)	36 (18-76)	1,480 (n/a)	20 ± 10	12 ± 2	37 ± 3	>12,500
KDU691	861 (699-1,063)	426 (277-638)	379 (n/a)	565 ± 9	354 ± 71	237 ± 57	150 ± 1
Methylene blue	40 (36-46)	18 (14-24)	95 (70-133)	15 ± 5	13 ± 1	12 ± 2	258 ± 29
Puromycin	261 (226-301)	103 (150-371)	949 (n/a)	122 ± 45	103 ± 39	110 ± 38	122 ± 48

IC_{50} values obtained in this study are derived from n=3 biological replicate dose response assays and 72-hour drug exposure.

Table S2. Oligonucleotides used for cloning and diagnostic PCRs on gDNA.

**Purpose**	**Oligonucleotide**	**Oligonucleotide sequence 5' → 3'**	**Name of fragment**
pHF_gC-ulg8	ulg8_sg1F	**tatt**ggtcctttatacgacacagg	sgt_*ulg8*
	ulg8_sg1R	**aaac**cctgtgtcgtataaaggacc	
pTRIX2-2A-RE9h	2a_F	*ctgtgcaagagttctagtg*GGTAGCGGAGAAGGAAGAGG	2a
	2a_R	*ttggcgtcctccatg*ccTGGATTTTCTTCTACATCTCCACAT	
pD_ulg8_re9h	5' box_F	*cgttggccgattcattaatg*CGATGAACATAACCTTGATACA	PCR fragment 1 (5' box)
	5' box_R	*ctccgctaccaTAAAAGAACGCATCC*TTATACT	
	2a-re9h_F	*ggatgcgttcttttatGGTAGCGGAGAAGG*AAGAGGAAGT	PCR fragment 2 (2a-re9h)
	2a-re9h_R	*catatggttgattgcaaTCAGATCTTGC*CGCCCTTCTTGG	
	3' box_F	*gcaagatctgaTTGCAATCAACCA*TATGACTTAAC	PCR fragment 3 (3' box)
	3' box_R	*cctcttcgctattacgccag*AACCCTAAAGCAAGATGTGTAATA	
	PCRA_F	*CTGGCGTAATAGCGAAGAGG*	PCR fragment 4 (pD backbone)
	PCRA_R	*CATTAATGAATCGGCCAACG*	
PCR on gDNA	ulg8 up_F2	AGCCTTGTCCTCAAAACTGG	5' integration
	re9h N_R	CTTGATGTTCTTGGCGTCCTCC	
	re9h C_F	GAAGGGCGGCAAGATCTG	3' integration
	ulg8 down_R2	ACATGGACTTACAACCAAAGG	
	ulg8 up_F2	AGCCTTGTCCTCAAAACTGG	wt locus or gene-edited locus
	ulg8 down_R2	ACATGGACTTACAACCAAAGG	

Names and sequences of oligonucleotides used in this study. Primer binding sites are highlighted in capital letters. Sequences involved in Gibson assembly are highlighted in italics. Single-stranded overhangs required for T4 DNA ligase-dependent cloning of the double-stranded sgRNA target sequence are highlighted in bold font.

Data file S1. List of compounds in the Epigenetics Screening Library (Cayman Chemical) and their inhibitory activity against mature stage V gametocytes (day 12) at 10 μM and 1 μM concentrations. RLU, relative luminescence units.

Data file S2. Comparison of the inhibitory activities of 101 shared compounds of the Epigenetics Screening Library (Cayman Chemical) screened at 1 μM concentration against mature stage V gametocytes (day 12) in this study and against stage IV/V gametocytes (day 10) by Vanheer et al.[67].

Data file S3. List of compounds targeting DNMTs, HKMTs and histone deacetylases and their inhibitory activity against mature stage V gametocytes (day 12) at 10 μM and 1 μM concentrations. RLU, relative luminescence units.

Data file S4. List of compounds in the kinase inhibitor library (SelleckChem, Enzo Life Sciences) and their inhibitory activity against mature stage V gametocytes (day 12) at 10 μM and 1 μM concentrations. RLU, relative luminescence units.

Data file S5. List of compounds in the Prestwick Chemical Library and their inhibitory activity against mature stage V gametocytes (day 12) at 10 μM and 1 μM concentrations. RLU, relative luminescence units.
