# Supplementary material for: An all-in-one pipeline for the in vitro discovery and in vivo testing of Plasmodium falciparum malaria transmission blocking drugs
Source: Nat Commun. 2025 Jul 25;16:6884. doi: 10.1038/s41467-025-62014-3 (PMC12297408; doi:10.1038/s41467-025-62014-3)
Supplement: Supplementary file 3 — Description of Additional Supplementary Files [file 41467_2025_62014_MOESM3_ESM.pdf]

Description of Additional Supplementary Files

File Name: Supplementary Data 1
Description: List of compounds in the Epigenetics Screening Library (Cayman Chemical) and their inhibitory activity against mature stage V gametocytes (day 12) at 10 µM and 1 µM concentrations. RLU, relative luminescence units.

File Name: Supplementary Data 2
Description: Comparison of the inhibitory activities of 101 shared compounds of the Epigenetics Screening Library (Cayman Chemical) screened at 1 µM concentration against mature stage V gametocytes (day 12) in this study and against stage IV/V gametocytes (day 10) by Vanheer et al.[67].

File Name: Supplementary Data 3
Description: List of compounds targeting DNMTs, HKMTs and histone deacetylases and their inhibitory activity against mature stage V gametocytes (day 12) at 10 µM and 1 µM concentrations. RLU, relative luminescence units.

File Name: Supplementary Data 4
Description: List of compounds in the kinase inhibitor library (SelleckChem, Enzo Life Sciences) and their inhibitory activity against mature stage V gametocytes (day 12) at 10 µM and 1 µM concentrations. RLU, relative luminescence units.

File Name: Supplementary Data 5
Description: List of compounds in the Prestwick Chemical Library and their inhibitory activity against mature stage V gametocytes (day 12) at 10 µM and 1 µM concentrations. RLU, relative luminescence units.
