# Supplementary material for: An all-in-one pipeline for the in vitro discovery and in vivo testing of Plasmodium falciparum malaria transmission blocking drugs
Source: Nat Commun. 2025 Jul 25;16:6884. doi: 10.1038/s41467-025-62014-3 (PMC12297408; doi:10.1038/s41467-025-62014-3)
Supplement: Supplementary file 9 — Reporting Summary [file 41467_2025_62014_MOESM9_ESM.pdf]

nature portfolio

Corresponding author(s): Nicolas M. B. Brancucci, Matthias Rottmann, Till S. Voss

Last updated by author(s): Jun 27, 2025

Reporting Summary

Nature Portfolio wishes to improve the reproducibility of the work that we publish. This form provides structure for consistency and transparency in reporting. For further information on Nature Portfolio policies, see our Editorial Policies and the Editorial Policy Checklist.

Statistics

For all statistical analyses, confirm that the following items are present in the figure legend, table legend, main text, or Methods section.

n/a	Confirmed	
☐	☒	The exact sample size (*n*) for each experimental group/condition, given as a discrete number and unit of measurement
☐	☒	A statement on whether measurements were taken from distinct samples or whether the same sample was measured repeatedly
☒	☐	The statistical test(s) used AND whether they are one- or two-sided<br>*Only common tests should be described solely by name; describe more complex techniques in the Methods section.*
☒	☐	A description of all covariates tested
☒	☐	A description of any assumptions or corrections, such as tests of normality and adjustment for multiple comparisons
☐	☒	A full description of the statistical parameters including central tendency (e.g. means) or other basic estimates (e.g. regression coefficient) AND variation (e.g. standard deviation) or associated estimates of uncertainty (e.g. confidence intervals)
☒	☐	For null hypothesis testing, the test statistic (e.g. *F*, *t*, *r*) with confidence intervals, effect sizes, degrees of freedom and *P* value noted<br>*Give P values as exact values whenever suitable.*
☒	☐	For Bayesian analysis, information on the choice of priors and Markov chain Monte Carlo settings
☒	☐	For hierarchical and complex designs, identification of the appropriate level for tests and full reporting of outcomes
☒	☐	Estimates of effect sizes (e.g. Cohen's *d*, Pearson's *r*), indicating how they were calculated

Our web collection on statistics for biologists contains articles on many of the points above.

Software and code

Policy information about availability of computer code

Data collection	Fluorescence microscopy was performed using a Leica Thunder 3D Assay fluorescence microscope (63x objective) equipped with a Leica K5 cMOS camera and Leica Application Suite X software (LAS X version 3.7.5.24914). Identical settings were used for both image acquisition and processing with Fiji (ImageJ2 version 1.54f).<br>High content fluorescence microscopy images were obtained with the ImageXpress Micro XLS widefield high content screening system (Molecular Devices) in combination with the MetaXpress software (version 6.5.4.532, Molecular Devices).<br>Luminescence images were acquired with an IVIS Lumina II in vivo imaging system (Caliper Life Sciences, PerkinElmer) at indicated exposure times. Data were analyzed and exported to a numerical format (counts) using Living Image (v4.7.2, Perkin Elmer).
Data analysis	All data have been analysed using Microsoft Excel 2016 or GraphPad Prism (version 8.2.1 or 10.0.1). Data from the high content screening system were subject to automated image analysis using the MetaXpress software (version 6.5.4.532, Molecular Devices). Figures were prepared using Adobe Illustrator (version 29.5.1).

For manuscripts utilizing custom algorithms or software that are central to the research but not yet described in published literature, software must be made available to editors and reviewers. We strongly encourage code deposition in a community repository (e.g. GitHub). See the Nature Portfolio guidelines for submitting code & software for further information.

Data

Policy information about availability of data

All manuscripts must include a data availability statement. This statement should provide the following information, where applicable:

- Accession codes, unique identifiers, or web links for publicly available datasets
- A description of any restrictions on data availability
- For clinical datasets or third party data, please ensure that the statement adheres to our policy

All data generated in this study are included in this published article and its Supplementary Information and Data files. Source data are provided with this paper. The Source data file contains the raw data underlying all graphs presented in the main manuscript and Supplementary Information file. Correspondence and requests for materials should be addressed to T.S.V, M.R. and N.M.B.B.

Research involving human participants, their data, or biological material

Policy information about studies with human participants or human data. See also policy information about sex, gender (identity/presentation), and sexual orientation and race, ethnicity and racism.

Reporting on sex and gender	n/a
Reporting on race, ethnicity, or other socially relevant groupings	n/a
Population characteristics	n/a
Recruitment	n/a
Ethics oversight	n/a

Note that full information on the approval of the study protocol must also be provided in the manuscript.

Field-specific reporting

Please select the one below that is the best fit for your research. If you are not sure, read the appropriate sections before making your selection.

[x] Life sciences [ ] Behavioural & social sciences [ ] Ecological, evolutionary & environmental sciences

For a reference copy of the document with all sections, see nature.com/documents/nr-reporting-summary-flat.pdf

Life sciences study design

All studies must disclose on these points even when the disclosure is negative.

Sample size	Sample sizes were chosen according to standards in the field. Most experiments were repeated at least three times independently as biological replicates, except for experiments conducted to confirm previously validated findings with independent cell lines, which were performed once or twice. The exact number of cells or animals analyzed in each experiment is provided in the Source data table (Figs 1c, 4a and 4c, 6a and 6c, 7, S2, S6 and S7) and figure captions and/or Methods section.<br>For microscopy-based quantification of gametocyte sex ratios and multiplication rates at least 200 parasites (infected red blood cells) have been scored per condition and experiment according to standards in the field (e.g. Filarsky et al., Science 2018, PMID: 29590075; Portugaliza et al., eLife 2020, PMID: 33084568; Bancells et al., Nat Microbiol 2019, PMID: 30478286).<br>For each gametocyte sample analysed by standard membrane feeding assays, 20 individual mosquitoes each from duplicate feeds were dissected according to standards in the field (e.g. Lensen et al., Trans R Soc Trop Med Hyg 1996, PMID: 8730302; Stone et al., Nat Commun 2018, PMID: 29422648).<br>For in vivo experiments, groups of two mice were used per experimental condition. Control groups with 3-4 mice were used per experiment.
Data exclusions	No data were excluded.
Replication	All experiments in this study were replicated successfully three times independently as stated in the figure legends, except for experiments conducted to confirm previously validated findings by a complementary approach or with independent cell lines:<br>- Primary screenings of chemical libraries were performed once, but hits were confirmed in dose response experiments.<br>- Mosquito feeding experiments via standard membrane feeding assays were performed once in duplicate feeds and with two animals per experimental group and 20 infected mosquitoes each.<br>- In vivo therapeutic efficacy of clinical drug candidates against NF54/iGP1_RE9Hulg8 stage V gametocytes in the NSG-PfGAM model were conducted once, but with at least two animals per dose group due to ethical and animal regulatory reasons.
Randomization	Mice were randomly assigned into experimental groups of n=2 animals.
Blinding	Blinding was either not relevant to this study because data were collected by automated readout methods (luminescence-based readout for parasite viability in vitro and in vivo, cell number quantification by high content imaging) or not possible because dose and control groups for

infected female NODscidIL2Rγnull (NSG) mice and mosquitoes were clearly defined and their handling requires highly specialized personnel.

Reporting for specific materials, systems and methods

We require information from authors about some types of materials, experimental systems and methods used in many studies. Here, indicate whether each material, system or method listed is relevant to your study. If you are not sure if a list item applies to your research, read the appropriate section before selecting a response.

Materials & experimental systems

n/a	Involved in the study
	x Antibodies
x	Eukaryotic cell lines
x	Palaeontology and archaeology
	x Animals and other organisms
x	Clinical data
x	Dual use research of concern
x	Plants

Methods

n/a	Involved in the study
x	ChIP-seq
x	Flow cytometry
x	MRI-based neuroimaging

Antibodies

Antibodies used

Primary antibody.
- rabbit α-Pfg377 (Alano et al., Mol Biochem Parasitol 1995; PMID: 8719156) was used at a 1:1,000 dilution.
Secondary antibody:
- Alexa Fluor 568-conjugated α-rabbit IgG (Molecular Probes #A11011) secondary antibody was used at 1:250 dilution

Validation

Primary antibody:
- The rabbit α-Pfg377 antibody has been published and validated (Alano et al., Mol Biochem Parasitol 1995; PMID: 8719156)
Secondary antibody:
- validation information for the commercially available Alexa Fluor 568-conjugated α-rabbit IgG (Molecular Probes #A11011) is available from the supplier.

Animals and other research organisms

Policy information about studies involving animals; ARRIVE guidelines recommended for reporting animal research, and Sex and Gender in Research

Laboratory animals

Female NODscidIL2Rγnull (NSG) mice (nine weeks old)

Wild animals

n/a

Reporting on sex

Only female NODscidIL2Rγnull (NSG) mice were used in this study as the engraftment protocol with human red blood cells was previously established only with female animals.

Field-collected samples

n/a

Ethics oversight

Animal studies performed at Swiss TPH were approved by the Veterinary Authorities of the Canton Basel-Stadt (permit no. 2992) based on Swiss cantonal (Verordnung Veterinäramt Basel-Stadt) and national regulations (the Swiss Animal Protection law, Tierschutzgesetz). All animal experiments performed at Radboud University were performed in accordance with the Dutch Experiments on Animals Act (Wod) and Directive 2010/63/EU from the European Union and the European ETS 123 convention, and were approved by the Radboud University Animal Welfare Body (IvD) and Animal Experiment Committee (RUDEC; 2015-0142) and the Central Authority for Scientific Procedures on Animals (CCD; AVD103002016424).

Note that full information on the approval of the study protocol must also be provided in the manuscript.

Plants

Seed stocks	n/a
Novel plant genotypes	n/a
Authentication	n/a
